# Supplementary material for: Implementation of Smart Triage combined with a quality improvement program for children presenting to facilities in Kenya and Uganda: An interrupted time series analysis
Source: PLOS Digit Health. 2025 Mar 10;4(3):e0000466. doi: 10.1371/journal.pdig.0000466 (PMC11892856; doi:10.1371/journal.pdig.0000466)
Supplement: S1 Table — (PDF) [file pdig.0000466.s001.pdf]

Table S1 Delayed treatments and reasons for delays

	Kenya			Uganda		
	Intervention site		Control site	Intervention site		Control site
**Variable**	**Baseline**	**Implementation**		**Baseline**	**Implementation**	
**Delayed treatments, n**	**1**	**4**	**92**	**275**	**176**	**24**
Out of stock, n (%)		2 (50%)	3 (3.26%)	118 (42.9%)	117 (66.78%)	2 (8.33%)
All HCW busy, n (%)[a]			87 (94.57%)	126 (45.81%)	41 (23.3%)	17 (70.83%)
**Other**						
Cannulation issue/collapsed veins, n (%)				21 (7.63%)	9 (5.11%)	3 (12.5%)
Blood transfusion, n (%)				1 (0.36%)		1 (4.17%)
Out of stock and all HCW busy, n (%) [b]				8 (2.9%)	6 (3.4%)	
Delay due to patient factors, n (%) [c]	1 (100%)		2 (2.17%)	1 (0.36%)	3 ((1.70%)	1 (4.17%)
No reason given, n (%)		2 (50%)	1 (1.09%)			

[a] All HCW busy: other reasons included in this: pharmacy closed, pharmacist not around, medical team in CME, delay in locating a ward, delay at pharmacy
[b] Out of stock & all HCW busy: other reason stated both as reasons for delay
[c] Delay due to patient factors: patient left, patient went to buy drugs and left, caretaker taking long time to make decisions, delays in treatment
